# The ADP-Ribosylation Factor 4d Restricts Regulatory T-Cell Induction via Control of IL-2 Availability

**DOI:** 10.3390/cells11172639

**Published:** 2022-08-25

**Authors:** Bernd Geers, Julia Hagenstein, Jessica Endig, Hanna Ulrich, Laura Fleig, Paulina Sprezyna, Julita Mikulec, Lukas Heukamp, Gisa Tiegs, Linda Diehl

**Affiliations:** 1 Institute of Experimental Immunology and Hepatology, University Medical Center Hamburg-Eppendorf, 20251 Hamburg, Germany; 2 Hamburg Center of Translational Immunology (HCTI), University Medical Center Hamburg-Eppendorf, 20246 Hamburg, Germany; 3 Institute of Experimental Medicine, University Hospital Bonn, 53127 Bonn, Germany; 4 Institute for Hematopathology, 22547 Hamburg, Germany

**Keywords:** regulatory T cell, Foxp3, pSTAT5, interleukin 2, Arl4d, immune regulation

## Abstract

Interleukin-2 is central to the induction and maintenance of both natural (nT_reg_) and induced Foxp3-expressing regulatory T cells (iT_reg_). Thus, signals that modulate IL-2 availability may, in turn, also influence T_reg_ homeostasis. Using global knockout and cell-specific knockout mouse models, we evaluated the role of the small GTPase ADP-ribosylation factor 4d (Arl4d) in regulatory T-cell biology. We show that the expression of Arl4d in T cells restricts both IL-2 production and responsiveness to IL-2, as measured by the phosphorylation of STAT5. *Arl4d*-deficient CD4 T cells converted more efficiently into Foxp3^+^ iT_reg_ in vitro in the presence of αCD3ε and TGFβ, which was associated with their enhanced IL-2 secretion. As such, *Arl4d*^−/−^ CD4 T cells induced significantly less colonic inflammation and lymphocytic infiltration in a model of transfer colitis. Thus, our data reveal a negative regulatory role for Arl4d in CD4 T-cell biology, limiting iT_reg_ conversion via the restriction of IL-2 production, leading to reduced induction of T_reg_ from conventional CD4 T cells.

## 1. Introduction

Tight control of the initiation and termination of immune responses is pivotal for the successful elimination of pathogens without causing collateral tissue damage or instigating autoreactivity. Regulatory T cells, expressing the signature transcription factor Foxp3, play an important role is this regulation. Natural Foxp3^+^ regulatory T cells (nT_reg_) that are generated during thymic T-cell differentiation are active in the periphery. Additionally, induced Foxp3^+^ T_reg_ (iT_reg_) can also be generated in the periphery from conventional CD4^+^ T cells in the context of inflammation. Such extra thymic T_reg_ differentiation is dependent on the presence of IL-2 and TGFβ [1]. Signaling via the high-affinity IL-2 receptor CD25, leading to the phosphorylation of STAT5, is essential for both nT_reg_ development [2] as well as the induction and stability of iT_reg_, as pSTAT5 is necessary for Foxp3 expression [3,4]. Both nT_reg_ and iT_reg_ express high levels of the IL-2 receptor CD25 but do not produce any significant amount of IL-2 themselves and are thus dependent on other cells as a source of IL-2 [5,6]. The high expression of and signaling via CD25 is not only necessary for T_reg_ induction, it is also pivotal for Treg suppressive function. IL-2 is locally sequestered by binding to CD25 on Foxp3^+^ T_reg_, which thereby reduces the availability of IL-2 for other IL-2-dependent immune cells, such as effector CD8 and CD4 T cells [7,8].

The ADP ribosylation factor 4d is part of the large family of ARF GTPases, mainly known for their role in membrane transport processes [9]. A functional role for Arl4d itself has been shown in actin remodeling [10], adipocyte development [11], neurite outgrowth [12], and microtubule growth [13], and Arl4d has known interaction partners (the cytohesin protein family) with immune function [14,15]. We could show that Arl4d expression is induced in CD8 T cells during priming by liver sinusoidal endothelial cells (LSEC), which is dependent on PD-L1/PD-1 signaling. In CD8 T cells, Arl4d expression interferes with Akt signaling and leads to the reduced development of both cytokine (IL-2 and IFNγ)-producing CD8^+^ effector T cells and short-lived effector cells (SLEC) in the context of viral infection in vivo [16].

Here, we report on the functional impact of Arl4d expression in CD4 T cells. Arl4d expression seems to be similarly regulated in CD4 T cells compared to CD8 T cells, in that it is downregulated upon TCR-mediated activation. Arl4d-deficiency in CD4 T cells results in an augmented secretion of IL-2, which is central to the augmented capacity of Arl4d-deficient CD4 T cells to convert into iT_reg_ in the presence of TGFβ. Although in vivo Arl4d deficiency in CD4 T cells leads to an increase in pSTAT5^+^ cells, this does not alter T_reg_ suppressive capacity. Thus, in summary, our data show that in CD4 T cells the expression of Arl4d is associated with an inhibition of IL-2 production, which ultimately leads to the repression of iT_reg_ conversion, and indicate that Arl4d has a regulatory function in both CD8 and CD4 T cells.

## 2. Materials and Methods

### 2.1. Animals

Arl4d^tm1a(EUCOMM)Wtsi^ [17], CD4-Cre × Arl4d^fl/fl^, Foxp3^YFP/iCre^ [18] × Arl4d^fl/fl^, C57BL/6J, Rag2^−/−^ [19], and OT-1 (C57BL/6-^Tg(TcraTcrb)1100Mjb^/J) [20] mice were bred and backcrossed in the animal facilities of the University Hospital Bonn and the University Medical Center Hamburg-Eppendorf, according to the Federation of European Laboratory Animal Science Association (FELASA) guidelines, and maintained under specific-pathogen-free (SPF) conditions. Arl4d^fl/fl^ mice were generated by crossing Arl4d^tm1a(EUCOMM)Wtsi^ with an flp-expressing deleter mouse line to cut out the Neomycin/LacZ cassette, leaving the floxed Arl4d allele intact. Hereafter, mice were crossed with the indicated Cre-expressing lines. Figure S2 shows the successful reduction of Arl4d mRNA expression in the relevant T-cell subsets in these mice. All mouse experiments were approved by the Behörde für Soziales, Familie, Gesundheit und Verbraucherschutz (approval code G30/15, G129/15, G19/17) and carried out according to the current existing guidelines on mouse experimentation. All efforts were made to minimize suffering.

### 2.2. CD4 T-Cell-Mediated Colitis in Rag2^−/−^ Mice

CD4 T cells were isolated from the spleen with the use of a naïve CD4^+^ T cell Isolation kit (Miltenyi Biotech, Bergisch Gladbach, Germany) according to the manufacturer’s recommendations. Cells were stained for CD4, CD25, and CD62L, after which sex-matched sorted splenic CD4^pos^CD25^neg^CD62L^high^ T cells were injected i.v. at 5 × 10^5^/mouse. Mice were weighed weekly, and at the indicated time, mice were sacrificed and the colon weight and length were measured. The colon was further processed for histological analysis.

### 2.3. In Vitro T_reg_, T_H_1, T_H_2, and T_H_17 Differentiation

CD4 T cells were isolated from the spleen with the use of a naïve CD4^+^ T cell Isolation kit (Miltenyi Biotech). Where indicated, these isolated CD4^+^ T cells were stained for CD25 and CD4, and CD4^+^CD25^high^ cells were sorted on a BD AriaIllu. For T_reg_ differentiation, CD4 T cells were plated onto CD3ε-coated plates (clone 145.C11, 1 μg/mL) in the presence or absence of 8 ng/mL muTGFβ (R&D Systems, Wiesbaden, Germany) and/or huIL-2 in the indicated concentrations. After 48 h, cells were harvested for flow cytometric analyses, and supernatants were analyzed for cytokine content by ELISA at the indicated times. For T_H_1, T_H_2, and T_H_17 differentiation, isolated splenic CD4^+^ T cells were cultured with use of CellXVivo^TM^ mouse T_H_1, T_H_2, and T_H_17 kits (R&D Systems) according to the manufacturer’s instructions.

### 2.4. Suppression Assay

Splenic CD4 T cells were isolated with a CD4 T cell isolation kit (Miltenyi Biotech) according to the manufacturer’s recommendations. Cells were stained for CD4 and CD25, and CD4^+^CD25^high^ cells were sorted on an Aria cell sorter (BD Biosciences). For the isolation of conventional CD4 T cells, splenocytes were labeled with 1 μM CFSE in warm PBS for 10′, after which 10% FCS was added to stop the reaction. CFSE-labeled CD4 T cells were then isolated with a CD4 T cell isolation kit as above. Sorted T_reg_ (CD4^+^ CD25^high^) were mixed with CFSE-labeled CD4^+^ T effector cells at the indicated ratios and cultured with or without anti-CD3ε/CD28 beads (ThermoFisher Scientific, Schwerte, Germany) in a 1:1 ratio to the CFSE^+^ T_eff_. After 72 h, the proliferation of the effector CD4 T cells was assessed by flow cytometry.

### 2.5. Ex Vivo pSTAT5 Detection

A single-cell suspension of splenocytes was incubated with the indicated amounts of IL-2 for 10 min at 37 °C and directly fixed in Roti Histofix 4% (Carl Roth, Karlsruhe, Germany) for 20′. After washing in FACS buffer (PBS, 0.5% FSC, and 0.02% NaN_3_), cells were permeabilized in 90% ice-cold methanol for 60′. pSTAT5 was stained using an alexafluor647-coupled anti-pSTAT5 antibody (C71E5, Cell Signalling Technologies, Leiden, the Netherlands) in combination with antibodies to CD8α, CD4, CD25, and Foxp3.

### 2.6. Flow Cytometry

Flow cytometry was performed using a Canto II or LSR II (BD Biosciences, Franklin Lakes, NJ, USA) and the data were analyzed using FlowJo software (v10, Becton Dickinson, Heidelberg, Germany). Antibodies were purchased from eBioscience (Thermofisher Scientific) or Biolegend (San Diego, CA, USA) unless otherwise stated. pSTAT5 antibodies and appropriate isotype control antibodies were from Cell Signalling Technology. A LIVE/DEAD Fixable Violet Dead Cell Stain kit (Thermofisher Scientific) was used to exclude dead cells. Intracellular transcription factor and cytokine staining was performed using a True-Nuclear^TM^ transcription buffer set (Biolegend). An anti-CD16/32 antibody (clone 2.4G2) was included in each staining at 10 μg/mL to block unspecific antibody binding via Fc receptors.

### 2.7. Histology

Liver and colon tissue were processed in routine paraffin embedding. Hematoxylin and Eosin (H&E) staining was performed using standard protocols. Sections were deparaffinized in Xylol and rehydrated in a descending range of EtOH and ddH_2_O. Inflammatory activity was scored in a four-tier scale of no–mild–moderate–severe active inflammation, defined as the infiltration of neutrophilic stromal, intra-epithelial, and luminal granulocytes estimated blinded on standard 5 μm H&E sections.

### 2.8. RNA Isolation, cDNA Synthesis, and Quantitative Real-Time PCR

mRNA was isolated from frozen pelleted cells using an RNeasy Mini kit (Qiagen, Hilden, Germany) following the manufacturer’s instructions. mRNA transcription into cDNA was performed using the High Capacity cDNA Reverse Transcription kit from Applied Biosystems (ThermoFisher Scientific). Quantitative RT-PCR (qPCR) was performed with exon-spanning primers, where possible, using the PowerUp SYBR Green Master Mix from Applied Biosystems (ThermoFisher Scientific) on a Viia QuantStudio 7 (ThermoFisher). The primers used were muArl4d forward: 5′-GACGAGTCGGTGTCTGGTTG-3′, muArl4d reverse: 5′-ACAAGCTGGGGTGTCTTCAG-3′, muIl2 forward: 5′-CCCAGGATGCTCACCTTCAAA-3′, muIl2 reverse: 5′-CCGCAGAGGTCCAAGTTCATC-3′, mu18S forward: 5′-CACGGCCGGTACAGTGAAAC-3′, mu18S reverse: 5′-AGAGGAGCGAGCGACCAAA-3′, muHprt forward: 5′-TGCTGACCTGCTGGATTACATT-3′, muHprt reverse: 5′-CTTTTATGTCCCCCGTTGACTG-3′, muEef1g forward: 5′-GTCTGTACCCTGTTGTGGCT-3′, muEef1g reverse: 5′-TCCCCCAAGATAGCCCTGAA-3′. Relative mRNA expression levels were calculated with the ΔCt method.

### 2.9. Methylation Analysis of Foxp3 TDSR

Genomic DNA from ex vivo-sorted CD4^+^CD25^high^ T cells was isolated using a GeneJet Genomic DNA purification kit (ThermoFisher Scientific), after which the DNA was digested with an EpiJet DNA methylation analysis kit (ThermoFisher Scientific) according to the manufacturer’s recommendations. Digested and undigested DNA was then used in a qPCR using primers specific for 5′-CCGG-3′ loci in the Foxp3 promotor: forward-1: 5′- TAGCACCCACATCACCCTACC-3′, reverse-1: 5′-CCACAGGTTTCGTTCCGAGAA-3′, forward-2: 5′-TTCCTCCTTGTTGCCGATGAAG-3′, reverse-2: 5′-CAACCTGAACTTGGCCAGATTTTT-3′.

### 2.10. Statistics

Statistical analysis was performed using Prism version 6.0h (Graphpad Software, San Diego, CA, USA). Student’s *t* or ANOVA tests were used to determine the statistical significance of the results. Data are depicted as means with the standard error of the mean (SEM), and *p*-values ≤ 0.05 were considered significant.

## 3. Results

### 3.1. Regulation of Arl4d mRNA in CD4 T Cells upon Activation

**Figure 1:**
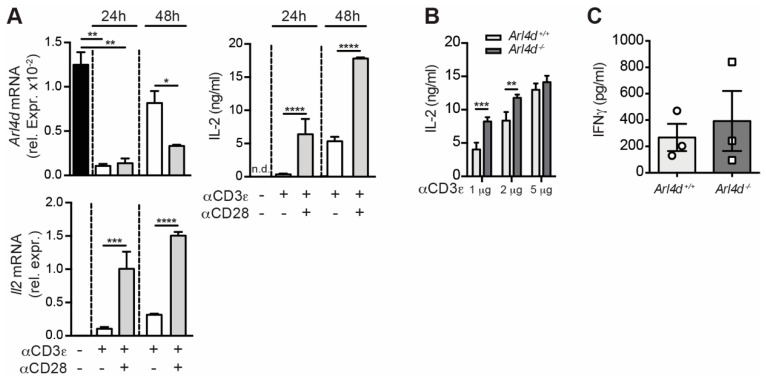
Regulation of Arl4d mRNA and protein in CD4 T cells. C57BL/6 splenic CD4^+^ T cells were stimulated with plate-bound αCD3ε (1–2 μg/mL) with (**A**) or without (**A**–**C**) soluble αCD28 antibodies (5 μg/mL) for the indicated times, after which (**A**) cells were harvested for RNA isolation and qPCR analysis of *Arl4d* mRNA and (**B**,**C**) supernatants were analyzed for their IL-2 or IFNγ contents by ELISA. (**A**,**B**) Representative of two independent experiments. (**C**) Cumulative data from three independent experiments. Statistical significance was calculated using ANOVA. * *p* ≤ 0.05, ** *p* ≤ 0.01, *** *p* ≤ 0.001, **** *p* ≤ 0.0001. n.d.: not detectable.

We have previously shown that, in CD8 T cells, *Arl4d* mRNA expression is downregulated upon activation and regulates IL-2 production and activity by these CD8 T cells [16]. We sought to analyze how *Arl4d* mRNA expression is regulated in CD4 T cells. To this end, we isolated wild-type CD4 T cells from C57BL/6 mice and stimulated them in vitro with plate-bound anti-CD3ε with or without soluble anti-CD28 (Figure 1A). Quantitative real-time PCR (qPCR) revealed that, similar to CD8 T cells, stimulation by αCD3 alone or in combination with anti-CD28 results in a rapid, profound, but also temporary loss of *Arl4d* mRNA expression. *Arl4d* mRNA was upregulated after 48 h compared to 24 h, which was the most efficient after αCD3ε stimulation alone. The correlation of the loss of *Arl4d* mRNA with an increase in *Il2* mRNA and IL-2 cytokine production was not as strong as we observed for CD8 T cells but was apparent at 48 h between αCD3ε and αCD3ε/αCD28 stimulated CD4 T cells. A direct effect of Arl4d expression on the ability to produce IL-2 was confirmed by the stimulation of *Arl4d*-deficient and -proficient CD4 T cells with αCD3ε, after which IL-2 production was significantly increased in the absence of Arl4d at lower αCD3ε concentrations (Figure 1B). This effect seems to be IL-2-specific, as for instance, IFNγ production by CD4 T cells was not changed due to Arl4d deficiency (Figure 1C). These data suggest that CD4 T cells, similar to CD8 T cells, are inhibited from producing IL-2 by Arl4d expression.

### 3.2. Arl4d-Deficient CD4 T Cells Are Less Pathogenic in a Model of Transfer Colitis

**Figure 2:**
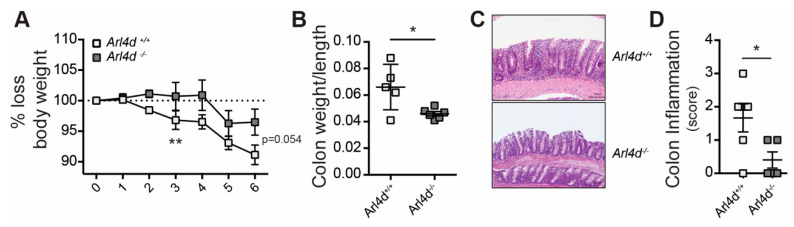
Arl4d deficiency does not aggravate CD4-mediated transfer colitis in Rag2^−/−^ mice. *Rag2*^−/−^ mice received 5 × 10^5^ sex-matched sorted splenic CD4^pos^CD25^neg^CD62L^high^ T cells from *Arl4d*^+/+^ or *Arl4d*^−/−^ mice. (**A**) Weight loss of the mice over time (as % of starting weight) in weeks. (**B**) Colon weight-to-length ratio at 7 weeks after T-cell transfer. (**C**) Representative H&E staining and (**D**) histological score of inflammation of the colons of mice transferred with either *Arl4d*^+/+^ or *Arl4d*^−/−^ CD4 T cells 7 weeks after T-cell transfer. Representative data of two experiments (*n* = 4–6/group). Statistical significance was calculated using ANOVA, * *p* ≤ 0.05, ** *p* ≤ 0.01.

In the absence of Arl4d, we previously showed that CD8 T cells gain effector function in the context of in vivo viral infection [16]. To test whether CD4 T cells were similarly affected by Arl4d deficiency, we used the model of transfer colitis by the adoptive transfer of CD25^neg^CD62L^high^ CD4 T cells from either *Arl4d*^+/+^ or *Arl4d*^−/−^ mice into *Rag2*-deficient mice. Mice that received CD25-depleted CD4 T cells proceeded to lose weight as expected, whereas control *Rag2*^−/−^ mice that did not receive any T cells gained weight. However, compared to the *Arl4d*^−/−^ CD4 T cells, the wild-type CD4 T cells induced more pronounced weight loss (Figure 2A). At 7 weeks, the calculated colon weight to length ratio revealed a significantly higher ratio in mice transferred with *Arl4d*^+/+^ T cells compared to mice that had received *Arl4d*^−/−^ T cells, demonstrating that more severe inflammation developed in those mice (Figure 2B). This could be confirmed by histological analysis, which revealed larger inflammatory infiltration of lymphocytes in H&E staining and the accompanying inflammation score (Figure 2C) due to the transfer of *Arl4d*^+/+^ CD4 T cells compared to *Arl4d*^−/−^ CD4 T cells, indicating that the expression of Arl4d in CD4 T cells augments colitis severity.

### 3.3. The Capacity of T Cells to Convert into Induced Regulatory T Cells (iT_reg_) Is Enhanced in the Absence of Arl4d

**Figure 3:**
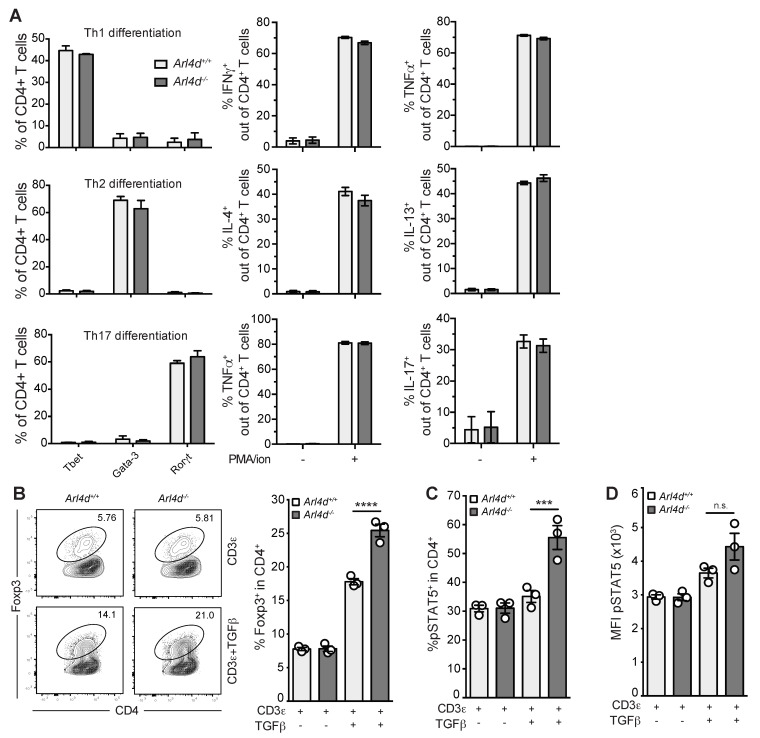
Arl4d controls regulatory T-cell conversion. (**A**) *Arl4d*^+/+^ or *Arl4d*^−/−^ splenic CD4^+^ T cells were differentiated into T_H_1, T_H_2, or T_H_17 cells using a commercial kit. After differentiation, CD4 T cells were stained intracellularly for the signature transcription factors T-bet (T_H_1), Gata-3 (T_H_2), and Rorγt (T_H_17). Additionally, CD4^+^ T cells were stimulated with PMA and ionomycin and stained intracellularly for the following cytokines: T_H_1: IFNγ and TNFα; T_H_2: IL-4 and IL-13; T_H_17: TNFα and IL-17. Bar graphs show the percentages of transcription-factor- or cytokine-expressing cells within the CD4^+^ T-cell population. (**B**–**D**) *Arl4d*^+/+^ or *Arl4d*^−/−^ splenic CD4^+^ T cells were stimulated with plate-bound αCD3ε (1 μg/mL) in the presence or absence mTGFβ (8 ng/mL) for 48 h, after which cells and supernatants were harvested and analyzed by flow cytometry and ELISA. (**B**) Representative dot plots of T_reg_ induction. Populations shown are gated on viable CD4^+^ cells. Bar graph also shows the percentage of Foxp3^+^ cells within the CD4^+^ T-cell population. (**C**) The percentage of pSTAT5^+^ cells within the CD4^+^ T-cell population. (**D**) MFI of pSTAT5 in pSTAT5^+^ CD4 T cells. Representative data of two (**A**) or three (**B**–**D**) experiments. Statistical significance was determined by Student’s *t* test, n.s.: not significant *** *p* ≤ 0.001, **** *p* ≤ 0.0001.

In general, it is thought that an imbalance between pro- and anti-inflammatory pathways is an important factor in the pathogenesis of inflammatory bowel disease [21]. In both humans and mouse models, there is evidence that various T-helper subsets can contribute to disease severity. In particular, an imbalance between T_H_17 cells and Foxp3^+^-induced regulatory T cells is associated with disease [22,23,24]. In order to explore whether the lack of Arl4d in CD4 T cells led to an altered capacity for development into T helper subsets, we performed in vitro T-cell differentiation assays for T_H_1, T_H_2, T_H_17, and iT_reg_ subsets. We found that Arl4d did not influence T_H_1, T_H_2, or T_H_17 differentiation, as measured by T-bet, Gata-3, and Rorγt expression and signature cytokine production (IFNγ, TNFα, IL-4, IL-13, and IL-17) after restimulation (Figure 3A). Additionally, Arl4d deficiency did not influence IL-2 production in fully differentiated T_H_1, T_H_2, and T_H_17 cells (Figure S1A), indicating that Arl4d primarily affects IL-2 production in undifferentiated CD4 T cells. However, in the absence of Arl4d, the conversion of naïve CD4 T cells into Foxp3^+^ regulatory T cells was markedly enhanced (Figure 3B). This was further associated with a significant increase in the percentage of pSTAT5-expressing CD4 T cells (Figure 3C), which is known to be pivotal for stable Foxp3 induction and expression in iT_reg_ [2], although the level of pSTAT5 on a per-cell basis does not seem to differ between Arl4d pro- and deficient T cells (Figure 3D). Thus, in CD4 T cells, the capacity to convert to Foxp3^+^ iT_reg_ is limited by Arl4d expression.

### 3.4. Enhanced T_reg_ Conversion in the Absence of Arl4d Correlates with Enhanced IL-2 Production in CD4 T Cells

**Figure 4:**
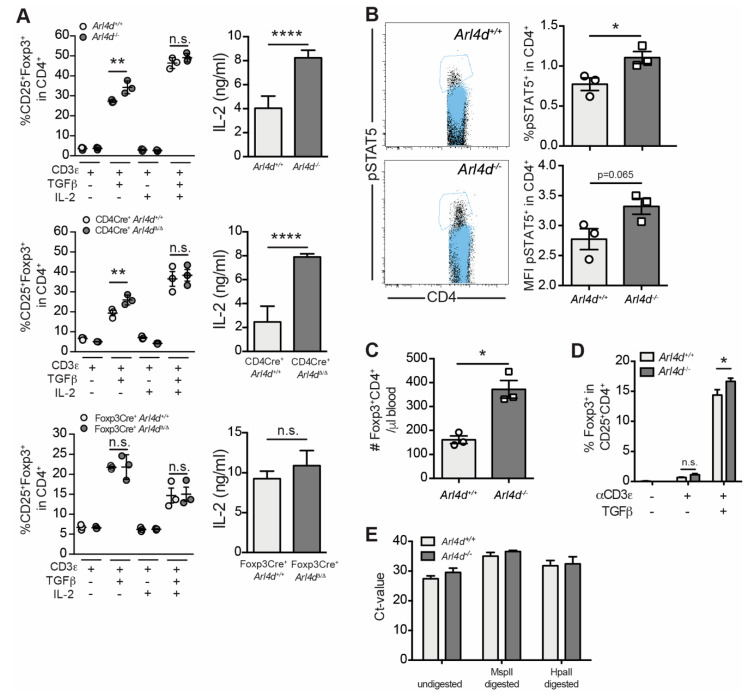
Differential capacity for IL-2 production associated with enhanced T_reg_ differentiation induced by Arl4d expression in CD4 T cells. Splenic CD4^+^ T cells from *Arl4d*^−/−^, CD4-Cre^+^*xArl4d*^Δ/Δ^, and Foxp3-Cre^+^*xArl4d*^Δ/Δ^ mice and their respective wild-type controls were stimulated with plate-bound αCD3ε (1 μg/mL) in the presence or absence of mTGFβ (8 ng/mL) or 100 IU/mL rhIL-2 for 48 h, after which cells and supernatants were harvested and analyzed by flow cytometry and ELISA. (**A**) Percentages of CD25^+^Foxp3^+^ cells within viable CD4^+^ T cells and the respective mIL-2 concentration (after αCD3ε stimulation) in the supernatant after 48 h of culture. (**B**) Ex vivo pSTAT5 staining of splenic CD4^+^ T cells from *Arl4d*^+/+^ or *Arl4d*^−/−^ mice. Dot plots show pSTAT5 staining (black dots) overlayed with isotype staining (blue dots). Bar graphs show the percentages and MFI of pSTAT5 in CD4^+^ and pSTAT5^+^ cells, respectively. (**C**) Absolute numbers (#) of Foxp3^+^CD4^+^ cells per μL of blood in *Arl4d*^+/+^ and *Arl4d*^−/−^ mice. (**D**) CD25^high^ depleted splenic CD4^+^ T cells were stimulated with plate-bound αCD3ε (1 μg/mL) in the presence or absence of mTGFβ (8 ng/mL) for 48 h, after which cells were stained for CD4, CD25, and Foxp3 (*n* = 3). (**E**) Undigested or digested genomic DNA from sorted splenic *Arl4d*^+/+^ and *Arl4d*^−/−^ CD4^+^CD25^high^ T cells was subjected to qPCR analysis of the Foxp3 promoter. Ct values of HpaI-digested samples that lie between the Ct values of undigested and MspII-digested samples indicate partial methylation (*n* = 2). Representative data of 3–4 (**A**) and 2 (**B**,**C**) experiments with *n* ≥ 3 (**B**,**C**). Statistical significance was determined by Student’s *t* test, * *p* ≤ 0.05, ** *p* ≤ 0.01, **** *p* ≤ 0.0001, n.s. = not significant.

TGFβ and IL-2 are pivotal signals for the conversion of CD4 T cells into Foxp3^+^ iT_reg_ [1]. Thus, based on our data, enhanced IL-2 production in the absence of Arl4d may be driving the observed enhanced iT_reg_ induction, as we did not add any external IL-2, only stimulated CD4 cells via the TCR in the presence of TGFβ (Figure 3B). Both CD4 T cells from global *Arl4d*^−/−^ and CD4-Cre^+^ × *Arl4d*^Δ/Δ^ mice (Figure S2) produced increased amounts of IL-2 upon stimulation with αCD3ε and converted into iT_reg_ more efficiently (Figure 4A).

However, in CD4 T cells from mice in which Arl4d was lacking only in Foxp3^+^ T_reg_, the capacity to produce higher amounts of IL-2 was lost, as was the capacity for enhanced conversion (Figure 4A). Moreover, the increased iT_reg_ induction in *Arl4d*^−/−^ and CD4-Cre^+^ × *Arl4d*^Δ/Δ^ CD4 T cells was lost when an external source of IL-2 was present (Figure 4A), suggesting that the altered IL-2 availability, due to the inherent capacity of *Arl4d*-deficient CD4 T cells to produce more IL-2, is promoting T_reg_ conversion in the absence of Arl4d. Consistent with this notion are the higher proportion of pSTAT5-expressing CD4 T cells (Figure 4B) and the higher numbers of circulating Foxp3^+^ CD4^+^ T_reg_ (Figure 4C) that we found in *Arl4d-*deficient mice. To validate that Arl4d-deficiency leads to increased conversion of CD4 T cells into Foxp3^+^ T_reg_, we set out to induce iT_reg_ from sorted CD25^high^ depleted CD4 T cells and could find a similar increase in Foxp3^+^ when stimulating these CD4 T cells with αCD3 in the presence of TGFβ (Figure 4D). However, the absence of Arl4d in T_reg_ did not influence the extent of methylation of the Foxp3 promotor. A qPCR analysis of genomic DNA from sorted CD25^high^CD4^+^ T cells from *Arl4d*^−/−^ and *Arl4d*^+/+^ animals digested with methylation-sensitive and -insensitive enzymes showed that, although the Foxp3 promoter is partially methylated, the levels of methylation were similar for Arl4d pro- and deficient Treg (Figure 4E). Thus, these data suggest that Arl4d functions to restrict IL-2-dependent iT_reg_ conversion but does not influence T_reg_ stability via Foxp3 promotor methylation.

### 3.5. Arl4d Expression Modulates IL-2 Receptor Signaling in CD4, CD8, and Foxp3 Regulatory T Cells but Does Not Modify T_reg_ Suppressive Function

IL-2 receptor signaling via the phosphorylation of STAT5 is pivotal for both iT_reg_ induction and T_reg_ function [2]. IL-2-mediated pSTAT5 signaling induces and maintains Foxp3 expression in iT_reg_ by preventing the demethylation of the CNS2 region in the Foxp3 locus [25], but it also influences T_reg_ with respect to their suppressive function [26]. We observed significantly higher proportions of pSTAT5^+^ in CD4 T cells from *Arl4d*-deficient mice (Figure 3B and Figure 4B) and a trend towards higher pSTAT5 levels on a per-cell basis (Figure 3D and Figure 4B). These effects could be due to the increased IL-2 availability due to augmented production by CD4 T cells. However, it is also possible that, due to *Arl4d* deficiency, signal transduction via the CD25 receptor, leading to pSTAT5 induction, is altered.

**Figure 5:**
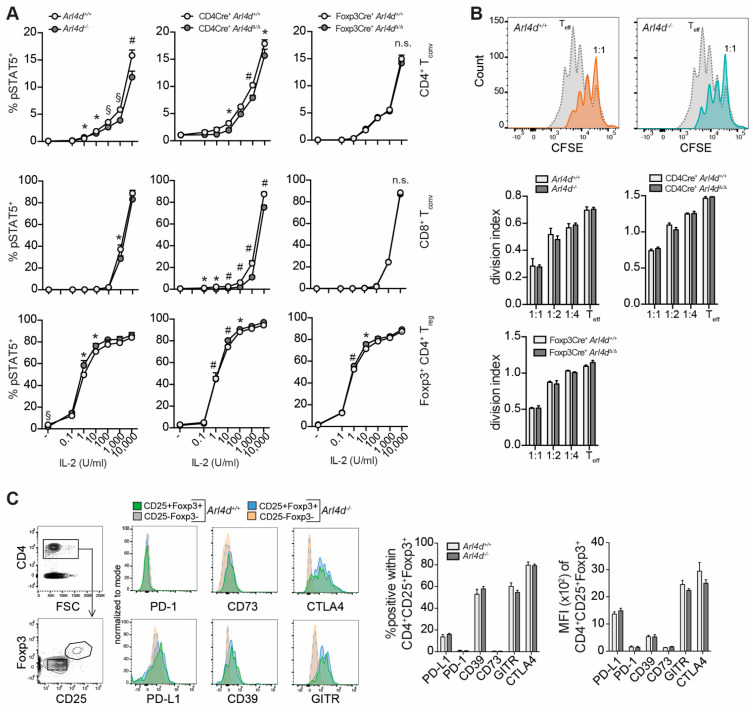
IL-2 receptor signaling and T_reg_ suppressive function in the presence or absence of Arl4d. (**A**) Splenocytes from the indicated mice were isolated and incubated for 10′ with rhIL-2 at the indicated concentrations. Subsequently, cells were fixed and stained for CD8α, CD4, CD25, Foxp3, and pSTAT5 antibodies. The percentages of pSTAT5^+^ cells were determined in CD8α^+^ T_conv_, CD4^+^ T_conv_, and CD4^+^Foxp3^+^ T_reg_ cells. (**B**) Splenic CD4^+^CD25^high^ T cells were sorted from mice as indicated. These were incubated with CFSE-labeled wild-type CD4^+^ T cells at the indicated T_reg_/T_eff_ ratios in the presence of αCD3ε/αCD28-coated beads. After 72 h, the proliferation of the CFSE^+^ CD4^+^ T cells was determined by flow cytometry. Histograms show an exemplary plot from CD4^+^ Teff incubated with either CD4^+^CD25^high^ T_reg_ from *Arl4d*^+/+^, *Arl4d*^−/−^ mice at a 1:1 ratio, or, as a control, CD4^+^ T_eff_ alone. Bar graphs show the division index. Representative data of 2–3 experiments. (**C**) Percentages and MFI of molecules associated with suppressive function in *Arl4d*^+/+^ or *Arl4d*^−/−^ CD4^+^CD25^+^Foxp3^+^ T_reg_ circulating in blood (*n* = 5). Statistical significance was determined by Student’s *t* test, * *p* ≤ 0.05, # *p* ≤ 0.01, § *p* ≤ 0.001, n.s.= not significant.

Thus, we set out to analyze pSTAT5 induction in T cells from Arl4d pro- and deficient mice (Figure 5A). Generally, the sensitivity of T lymphocytes to IL-2/CD25 signaling, as measured by pSTAT5 induction, was highest in Foxp3^+^ CD4^+^ T_reg_, followed by CD4^+^ T cells and CD8^+^ T cells. By using ex vivo splenocytes from *Arl4d*^−/−^, CD4-Cre^+^ × *Arl4d*^Δ/Δ^, and Foxp3-Cre^+^ × *Arl4d*^Δ/Δ^, we can conclude that the deficiency of Arl4d changes the sensitivity of all three types of T lymphocytes to IL-2 receptor signaling, as demonstrated by consistent, significant, albeit small, changes in the proportion of cells expressing pSTAT5 (Figure 5A). The marginally increased pSTAT5 signaling in T_reg_, however, does not seem affect T_reg_ function, although this has been described [26], as we could not find differences in the suppressive function of ex vivo-sorted CD25^high^ CD4^+^ T_reg_ in a classical suppression assay towards the proliferation of conventional effector CD4^+^ T cells (Figure 5B), nor did we detect differences in the expression of molecules (PD-1, PD-L1, CD39, CD73, GITR, and CTLA4) associated with suppressive function due to Arl4d deficiency in both ex vivo-isolated T_reg_ (Figure 5C) and in vitro-induced T_reg_ (Figure S1B).

## 4. Discussion

In this study we set out to evaluate the impact of the expression of the ARF-like GTPase Arl4d on CD4 T-cell function. We have previously shown that Arl4d plays an important role in the control of CD8 T-cell effector function via the regulation of the PI3K pathway, regulating the development of IL-2/IFN γ-producing short-lived effector cells (SLEC) [16]. Here, we show that in CD4 T cells Arl4d also plays a regulatory role in controlling cytokine production, most prominently IL-2 (Figure 1). Similar to CD8 T cells, CD4 T cells lose *Arl4d* mRNA expression after T-cell activation, which is more pronounced under full T-cell activation conditions (αCD3ε plus αCD28 stimulation versus αCD3ε alone), and Arl4d deficiency results in significantly more IL-2 secretion by CD4 T cells. This increased cytokine production by *Arl4d*^−/−^ CD4 T cells, however, does not exacerbate CD4 T-cell-driven colitis but rather protects from extensive tissue inflammation and cell infiltration (Figure 2). This was associated with an enhanced capacity of *Arl4d*^−/−^ CD4 T cells to convert into CD25^+^Foxp3^+^ iT_reg_ (Figure 3). Although IL-2 has been shown to fine-tune T-helper differentiation, ranging from promoting T_H_1 and T_H_2 differentiation to inhibiting T_H_17 differentiation [27], Arl4d only seems to affect T_reg_ conversion and does not affect the efficiency of CD4 T cells differentiate into T_H_1, T_H_2, or T_H_17.

In the absence of Arl4d, CD4 T cells develop more prominently into Foxp3-expressing cells, suggesting Arl4d functions to limit T_reg_ conversion in wild-type cells. As iT_reg_ induction relies on the presence of a TCR trigger (αCD3ε), TGFβ, and IL-2, in theory Arl4d may modulate one or more of these signaling pathways. We focused on the effect of Arl4d on IL-2 production and availability, firstly because *Arl4d*^−/−^ CD4 T cells produce more IL-2 upon TCR triggering and secondly because we found higher numbers of pSTAT5^+^ CD4 T cells in in vitro iT_reg_ conversion settings as well as ex vivo in CD4 T cells from *Arl4d*^−/−^ animals, indicating altered IL-2 signaling (Figure 3C and Figure 4B). Together with the observed tendency for higher pSTAT5 levels per cell in the absence of Arl4d, the question remained whether the increased IL-2 production by *Arl4d*^−/−^ CD4 T cells was leading to increased iT_reg_ conversion or whether the absence of Arl4d enhanced IL-2 receptor signaling in CD4 T cells. By titrating IL-2 and analyzing STAT5 phosphorylation in ex vivo splenocytes, the lack of Arl4d only marginally changed IL-2 receptor signaling in CD4 T cells as well as CD8 T cells and Foxp3^+^ T_reg_ (Figure 5), decreasing it in CD4 and CD8 and increasing it in Foxp3^+^ cells. Thus, enhanced sensitivity towards IL-2 is not a likely cause for the increased iT_reg_ conversion in *Arl4d*^−/−^ CD4 T cells. However, only when Arl4d deficiency is present in the CD4 T-cell compartment (*Arl4d*^−/−^ and CD4-Cre^+^ × *Arl4d*^Δ/Δ^ but not Foxp3-Cre^+^ × *Arl4d*^Δ/Δ^) is there enhanced iT_reg_ conversion and enhanced IL-2 production (Figure 4A). Thus, Arl4d restricts iT_reg_ conversion in CD4 T cells by limiting IL-2 production by conventional CD4 T cells. This is underpinned by the fact that the addition of external IL-2 bypasses the augmented iT_reg_ conversion completely. Thus, when the ability to produce larger amounts of IL-2 is lost in CD4 T cells, so are the higher numbers of induced T_reg_. As observed in vivo, the cellular source of IL-2 for T_reg_ induction/maintenance has been shown to reside solely in T cells, not in IL-2-producing B cells and dendritic cells [5]. The enhanced capacity of *Arl4d*^−/−^ CD4 T cells to produce IL-2 most likely counts for the higher observed numbers of Foxp3^+^ and pSTAT5-expressing CD4 T cells found in *Arl4d*^−/−^ mice, although the absence of Arl4d seems to marginally increase CD25 signaling, as shown by higher pSTAT5 levels in CD4, CD8α, and Foxp3^+^ CD4 T cells.

IL-2 signaling via CD25 leads to the phosphorylation of STAT5, which in turn promotes Foxp3 expression via binding to the demethylated conserved noncoding sequence 2 (CNS2) in the promotor/enhancer region of the Foxp3 locus [25,28]. Not only is the expression of Foxp3 essential for maintaining the T_reg_ phenotype, signaling via STAT5 is also involved in the suppressive function of T_reg_ [26]. Our data, however, suggest that although Arl4d controls STAT5 signaling strength (Figure 5) in T_reg_, leading to higher methylation of the Foxp3 promoter, it does not affect the suppressive function on a per-cell basis. Both the unchanged capacity of ex vivo-sorted *Arl4d*^−/−^ Foxp3^+^ T_reg_ to inhibit CD4 T-cell proliferation and the unchanged expression of suppressive molecules, such as CTLA4, CD39, and CD73, on *Arl4d*^−/−^ T_reg_ indicate that the changed IL-2 availability is the main cause of the observed effects on T_reg_. We have, however, not analyzed the suppressive function of Arl4d-deficient T_reg_ toward CD8 T cells, which may be inhibited in a different manner than CD4 T cells [26]. Additionally, although the combination of enhanced IL-2 availability and the small but significant enhanced sensitivity to signaling via CD25 in *Arl4d*^−/−^ T_reg_ may influence the methylation of the Foxp3 promoter and thus the stability of Foxp3^+^ CD4 T cells [26,29], we could not find such an influence due to Arl4d deficiency. As the control of IL-2 production and availability is central to T_reg_ induction, function, and homeostasis, defining molecular cues that regulate this is important to understand T_reg_ biology. Here, we show that the small GTPase Arl4d functions to limit IL-2 production in CD4 T cells, which directly impacts on the capacity of those CD4 T cells to convert into T_reg_.

## Data Availability

The data presented in this study are available on request from the corresponding author upon reasonable request.

## Supplementary Materials

The following supporting information can be downloaded at: https://www.mdpi.com/article/10.3390/cells11172639/s1, Figure S1: (A) *Arl4d*^+/+^ or *Arl4d*^−/−^ splenic CD4^+^ T cells were differentiated into T_H_1, T_H_2, or T_H_17 cells using a commercial kit. After differentiation, CD4 T cells were stimulated with PMA and ionomycin and stained intracellularly for IL-2. (B) Expression of various surface markers associated with T_reg_ suppressive activity in in vitro-induced Foxp3^+^ CD4 T cells from *Arl4d*^+/+^, *Arl4d*^−/−^, CD4-Cre^+^*xArl4d*^+/+^, CD4-Cre^+^*xArl4d*^Δ/Δ^, Foxp3-Cre^+^*xArl4d*^+/+^, and Foxp3-Cre^+^*xArl4d*^Δ/Δ^ animals; Figure S2: Successful knockdown of *Arl4d* mRNA in CD4-Cre × *Arl4d^fl/fl^* and Foxp3-Cre × *Arl4d* ^fl/fl^ mice. Splenic CD4^+^ T cells and Foxp3^+^ (YFP^pos^) CD4^+^ T cells were isolated by magnetic beads and flow cytometric sorting, after which RNA was isolated for qPCR of Arl4d mRNA. As a control, splenic CD4^+^ T cells from global Arl4d-deficient mice were used. Statistical significance was determined by Student’s *t* test, ** *p* ≤ 0.01, **** *p* ≤ 0.0001, n.d.= not detectable.
