# Supplementary material for: The ADP-Ribosylation Factor 4d Restricts Regulatory T-Cell Induction via Control of IL-2 Availability
Source: Cells. 2022 Aug 25;11(17):2639. doi: 10.3390/cells11172639 (PMC9454872; doi:10.3390/cells11172639)
Supplement: Supplementary file 1 [file cells-11-02639-s001.zip › cells-1768137-supplementary.pdf]

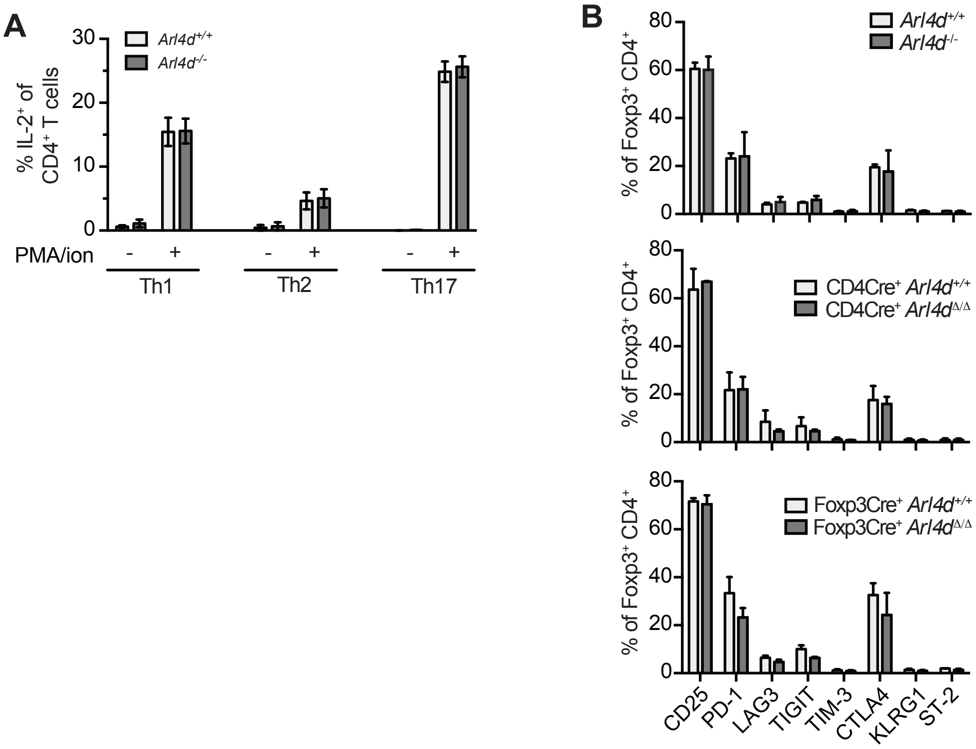

Figure S1: (A) *Arl4d*$^{+/+}$ or *Arl4d*$^{-/-}$ splenic CD4$^+$ T cells were differentiated into T_H1, T_H2, or T_H17 cells using a commercial kit. After differentiation, CD4 T cells were stimulated with PMA and ionomycin and stained intracellularly for IL-2. (B) Expression of various surface markers associated with T_{reg} suppressive activity in *in vitro*-induced Foxp3$^+$ CD4 T cells from *Arl4d*$^{+/+}$, *Arl4d*$^{-/-}$, CD4-Cre$^+$x*Arl4d*$^{+/+}$, CD4-Cre$^+$x*Arl4d*$^{\Delta/\Delta}$, Foxp3-Cre$^+$x*Arl4d*$^{+/+}$, and Foxp3-Cre$^+$x*Arl4d*$^{\Delta/\Delta}$ animals.

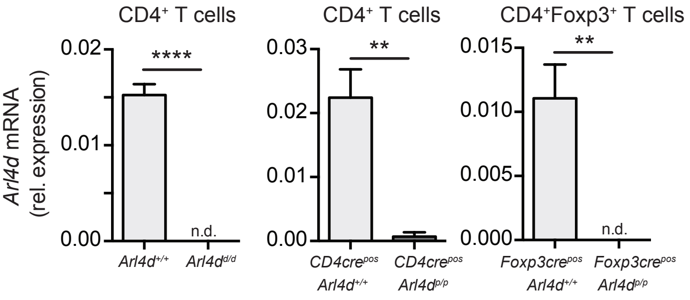

Figure S2: Successful knockdown of *Arl4d* mRNA in CD4-Cre x *Arl4d*$^{fl/fl}$ and Foxp3-Cre x *Arl4d* $^{fl/fl}$ mice. Splenic CD4^{+} T cells and Foxp3^{+} (YFP^{pos}) CD4^{+} T cells were isolated by magnetic beads and flow cytometric sorting, after which RNA was isolated for qPCR of *Arl4d* mRNA. As a control, splenic CD4^{+} T cells from global *Arl4d*-deficient mice were used. Statistical significance was determined by Student's *t* test, ** $p \leq 0.01$, **** $p \leq 0.0001$, n.d.= not detectable.
